# Research monitoring practices in critical care research: a survey of current state and attitudes

**DOI:** 10.1186/s12874-022-01551-7

**Published:** 2022-03-21

**Authors:** Renate Le Marsney, Tara Williams, Kerry Johnson, Shane George, Kristen S. Gibbons

**Affiliations:** 1 Paediatric Critical Care Research Group, Child Health Research Centre, The University of Queensland, Brisbane, QLD Australia; 2 Paediatric Intensive Care Unit, Queensland Children’s Hospital, Children’s Health Queensland, Brisbane, QLD Australia; 3 School of Medicine and Menzies Health Institute Queensland, Griffith University, Southport, Australia; 4 Gold Coast University Hospital, Southport, Australia

**Keywords:** Clinical trial, Monitoring, Risk-based monitoring

## Abstract

**Background/Aims:**

In 2016, international standards governing clinical research recommended that the approach to monitoring a research project should be undertaken based on risk, however it is unknown whether this approach has been adopted in Australia and New Zealand (ANZ) throughout critical care research. The aims of the project were to: 1) Gain an understanding of current research monitoring practices in academic-led clinical trials in the field of critical care research, 2) Describe the perceived barriers and enablers to undertaking research monitoring.

**Methods:**

Electronic survey distributed to investigators, research co-ordinators and other research staff currently undertaking and supporting academic-led clinical trials in the field of critical care in ANZ.

**Results:**

Of the 118 respondents, 70 were involved in the co-ordination of academic trials; the remaining results pertain to this sub-sample. Fifty-eight (83%) were working in research units associated with hospitals, 29 (41%) were experienced Research Coordinators and 19 (27%) Principal Investigators; 31 (44%) were primarily associated with paediatric research. Fifty-six (80%) develop monitoring plans with 33 (59%) of these undertaking a risk assessment; the most common barrier reported was lack of expertise. Nineteen (27%) indicated that centralised monitoring was used, noting that technology to support centralised monitoring (45/51; 88%) along with support from data managers and statisticians (45/52; 87%) were key enablers. Coronavirus disease-19 (COVID-19) impacted monitoring for 82% (45/55) by increasing remote (25/45; 56%) and reducing onsite (29/45; 64%) monitoring.

**Conclusions:**

Contrary to Good Clinical Practice guidance, risk assessments to inform monitoring plans are not being consistently performed due to lack of experience and guidance. There is an urgent need to enhance risk assessment methodologies and develop technological solutions for centralised statistical monitoring.

**Supplementary Information:**

The online version contains supplementary material available at 10.1186/s12874-022-01551-7.

## Introduction

Undertaking clinical research is vital to discovering new treatments in the ongoing quest to enhance patient care; in particular, clinical trials provide the highest level of evidence when evaluating effectiveness of a proposed treatment or therapy. The International Council for Harmonisation of Technical Requirements for Pharmaceuticals for Human Use Good Clinical Practice (ICH-GCP) guidelines specify that monitoring of clinical trial data is essential in undertaking high quality clinical trials; in fact, it is well accepted that if there is 10% or more missing or incorrect data, the resulting analysis may be unreliable [1, 2], highlighting the importance of such a process. While the ICH-GCP recommendations are non-binding, they are a well-accepted international standard that has been endorsed across many jurisdictions [3–7].

In 2016 an addendum to ICH-GCP E6 was released, updating the guidance relating to data monitoring to incorporate an approach that is “… *systematic, prioritized, risk-based”* [8], a shift from the previous guidance that often resulted in source data verification (SDV) of 100% of data points [9, 10]. However, despite the estimate that 25–40% of the total cost of a clinical trial represents monitoring-related costs [11, 12], there is limited evidence to support the methodologies pertaining to monitoring practices undertaken for clinical trials [13, 14]. Acknowledging that 100% SDV does not have the same impact on enhanced data quality as once thought [10, 15–17], it has been estimated that a move towards risk-based monitoring has the potential to reduce monitoring costs by up to 35% for large studies [18], highlighting the importance of the uptake of this recommendation, particularly for academic-led clinical trials with limited resources. This is particularly relevant to critical care research where trials are rarely funded by industry; a recent meta-epidemiologic study reported that of 568 trials, only 88 (15.5%) were funded by industry, with a further 73 (12.9%) co-funded by industry and non-profit funding [19].

The uptake of a risk-based monitoring approach has been documented in the United Kingdom, where a survey of clinical trial units (CTUs) reported that all CTUs planned their monitoring approach following an assessment of risk for clinical trials of an investigational medicinal product (CTIMPs), and 91% for non-CTIMPs [20]. More broadly across Europe, 41% of clinical research associates reported working within a risk-based monitoring model [21], however, in Ireland only 21% of respondents had performed RBM in a clinical trial setting [22]. A network of Canadian paediatric clinical trial centres has outlined a centralised approach to risk-based monitoring [23]. However, it is largely unknown if the risk-based approach to monitoring has been widely implemented in Australian and New Zealand (ANZ) research sites.

This study sought to gain an understanding of current research monitoring practices in academic-led clinical trials in the field of critical care research in ANZ, including perceived barriers and enablers to undertaking research monitoring, and identify if there were characteristics of research units or research staff that resulted in a higher rates of monitoring plan development and use of risk assessments.

## Methods

An electronic survey was distributed to clinicians and researchers in the field of critical care in ANZ. Inclusion criteria for participants were: undertaking or supporting clinical research in a critical care setting; been involved in undertaking or supporting a clinical trial in the past five years; and working in Australia or New Zealand. There were no exclusion criteria. The study received ethical approval from The University of Queensland Human Research Ethics Committee (approval number: 2020001788).

The survey was developed following a review of the literature. A limited number of surveys examining similar topics have been published [20, 22, 24, 25]; we reviewed these surveys and included questions relevant to the ANZ critical care setting, and specifically, academic-led clinical trials. Additionally, we developed questions relevant to our setting, and particularly in response to the coronavirus disease-19 (COVID-19) pandemic and the impact it has had on undertaking monitoring.

Following the development of the survey by the research team, we piloted the survey and assessed the face validity by requesting a number of staff in our research unit in different roles and with differing levels of experience undertake the survey. The survey was refined based on this feedback; the final survey is available in Supplementary Material S1. The survey was implemented in the REDCap electronic data capture tool hosted at The University of Queensland [26, 27].

The survey was distributed via the professional groups who represent the majority of critical care researchers in ANZ; Australian and New Zealand Intensive Care Society (ANZICS) Clinical Trials Group, Paediatric Study Group, Paediatric Intensive Care Research Coordinators Interest Group, Intensive Care Research Coordinators Interest Group, Australasian College for Emergency Medicine (ACEM) and Paediatric Research in Emergency Departments International Collaborative (PREDICT). The survey was emailed via either a specific survey invitation, or contained within a regular email newsletter communication, to the distribution lists of these networks. An email reminder was sent through each of the professional networks 4–6 weeks following the initial invitation. In addition to distribution through these networks, respondents were also asked to forward the survey on to any other research staff working in critical care research that are not members of these groups, and the survey was promoted at scientific meetings of these professional groups which occurred during the survey window. The first page of the survey contained screening questions and if respondents did not meet the eligibility criteria they were thanked for their time and the complete survey was not shown. Completion and submission of the survey was taken as implied consent. There were no incentives to participate.

### Statistical analysis plan

Data are presented as number and proportions. As the survey questions were not mandatory, there are varying levels of missing data; the denominator is also presented where necessary. Free-text responses were reviewed by the study team and allocated to themes, and are presented descriptively where required. Bivariate analysis was undertaken to explore the association between respondent characteristics and two key outcomes: 1) development of a monitoring plan, and 2) use of a risk assessment when developing a monitoring plan. A series of bivariate logistic regression models were generated for each of these two outcomes with the following respondent characteristics investigated: type of institution (hospital, university, other); country (Australia, New Zealand); primary patient group (paediatric, adult); type of research undertaken (academic-led, industry-sponsored, international, single-site, multi-site); trial role (Research Co-ordinator, Principal Investigator, Other); trial experience (≤ 6 years, > 6 years) and clinical trial training undertaken (GCP – face-to-face, GCP – online, monitoring specific). Odds ratios and 95% confidence interval are presented alongside descriptive statistics. Analyses were undertaken in StataSE version 16.0 (StataCorp Pty Ltd, College Station, Texas).

## Results

The survey link was sent to 9,604 email addresses through the mailing lists of the professional bodies, incorporating recipients across clinical, research, teaching and other disciplines; many respondents were on one or more of these mailing lists, therefore this number over-represents the potential respondent group. One hundred and fifty-four responses were received. Twenty responses were excluded as they did not meet the eligibility criteria for the survey or did not provide a minimum dataset for analysis. A further 16 responses were excluded where the respondent was not involved in critical care research. One hundred and eighteen responses were included in the final analysis (Supplementary Table 1).

Seventy-one (60.2%) respondents were from research units that coordinate academic led clinical trials. One respondent provided no further information, therefore the following results for monitoring practices and enablers and barriers to different aspects of monitoring pertain to these 70 respondents unless otherwise specified. Respondents were predominantly experienced research co-ordinators working in Australian hospital-based research units; 56% in the adult setting, with 71% having completed face-to-face GCP training, and 37% completing monitoring specific training (Table 1).

**Table 1:** Characteristics of survey respondents who are involved in co-ordination of academic-led clinical trials

Characteristic	*N* = 70 n (%)
***Research Unit***	
Research Unit Affiliation*	
Hospital	58 (83)
University	36 (51)
Other	5 (7)
* Country*	
Australia	62 (89)
New Zealand	8 (11)
* Patient Group*	
Paediatric	31 (44)
Adult	39 (56)
Additional Area/s of Research*	
Emergency Medicine	33 (47)
Intensive Care Medicine	33 (47)
Anaesthetic Medicine	25 (36)
Operating Room Medicine	13 (19)
Other	9 (13)
***Respondent***	
Clinical Trial Types*	
Academic-led	65 (93)
Industry-led	33 (47)
International	62 (89)
Single-site	49 (70)
Multi-site	67 (96)
*Primary Role*	
Principal Investigator	19 (27)
Research Coordinator	29 (41)
Research Nurse	9 (13)
Study Monitor	4 (6)
Data Manager	2 (3)
Pharmacist	1 (1)
Other	6 (9)
*Years in Clinical Trials*	
< 1 year	1 (1)
1–3 years	18 (26)
4–6 years	14 (20)
> 6 years	37 (53)
*Highest Level of Education*	
Undergraduate Degree	6 (9)
Postgraduate Degree	48 (69)
Doctorate	15 (21)
Missing	1 (1)
Clinical Trials Training*	
Good Clinical Practice – Face-to-face	50 (71)
Good Clinical Practice – Online	63 (90)
Monitoring specific training	26 (37)
Other	12 (17)
None of the above	1 (1)

^*^one or more responses could be selected

### Development of monitoring plans

Eighty percent (56 of 70) developed monitoring plans always or some of the time, primarily before the trial has commenced recruitment (35 of 56; 63%). Forty-seven percent (33 of 56) use a risk assessment to inform the monitoring approach and where a risk assessment is conducted, the main study aspects assessed for their associated risk were reporting of adverse events (29 of 33; 88%), compliance with the study protocol (27 of 33; 82%) and completion of completeness and accuracy of primary and secondary endpoint data (25 of 33; 76%). A template or standard operating procedure is used to conduct the risk assessment 76% (25 of 33) of the time. These risk assessment tools are primarily developed by the research unit (26 of 33; 79%) and mostly use a method of staff judgement to assess the risk associated with each study aspect (22 of 33; 67%). The risk assessment is revisited throughout the course of the study 79% (26 of 33) of the time. The most common reasons for conducting a risk assessment are to improve patient safety and data accuracy, while lack of expertise is the most common barrier (Table 2).

**Table 2:** Enablers and barriers related to the conduct of a risk assessment for informing a monitoring plan

Enablers for those who conduct a risk assessment *N* = 33		Barriers for those who do not always conduct a risk assessment *N* = 17	
**Enabler**	**n (%)**	**Barrier**	**n (%)**
To improve patient safety	29 (88)	Do not have the expertise to perform a risk assessment	10 (59)
To improve data accuracy	29 (88)	It is too time consuming	5 (29)
To fulfil GCP requirements	24 (73)	Other	4 (24)
To improve efficiency and objectivity of monitoring	20 (61)	It is not a requirement of the Sponsor	4 (24)
To determine a schedule for onsite monitoring visits	12 (36)	Not sure	4 (24)
To fulfil Sponsor requirements	10 (30)	It is too expensive	2 (12)
To reduce monitoring costs	10 (30)	It is not a GCP requirement	0
Other	0	It will not improve patient safety	0
Not sure	0	It will not improve efficiency or objectivity of monitoring	0

### Onsite monitoring

**Fig. 1:**
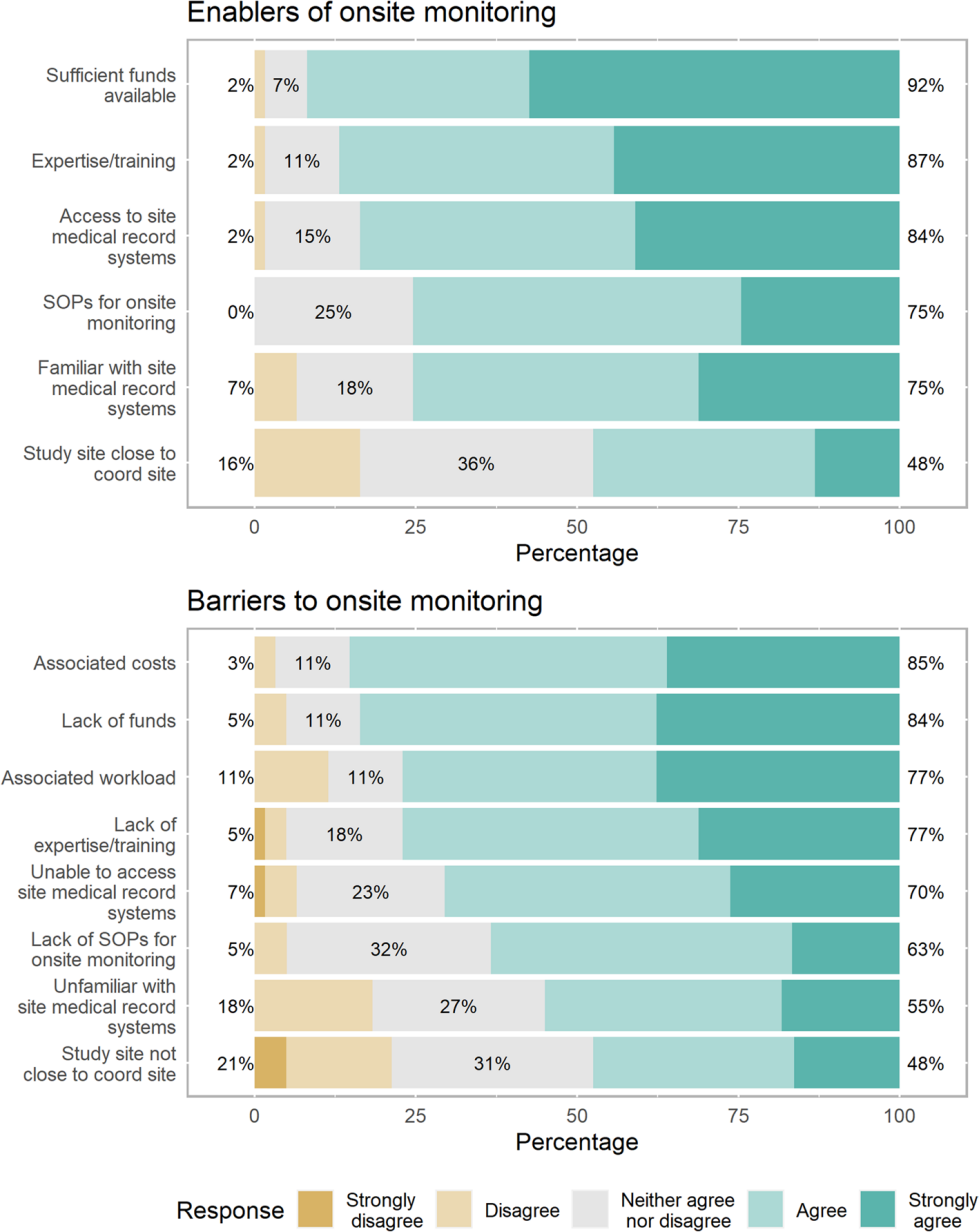
Perceived enablers and barriers to performing onsite monitoring

Onsite monitoring visits are performed by the respondent’s research unit 78% (49 of 63) of the time. Where onsite monitoring visits are conducted, the main study aspects assessed are compliance with eligibility (45 of 49; 92%), compliance with the informed consent process (44 of 49; 90%) and source data verification (44 of 49; 90%). The frequency of onsite monitoring visits is most commonly determined by the monitoring plan in the protocol (29 of 49; 59%) and the study design (25 of 49; 51%). Where specific triggers determine the frequency of onsite visits (14 of 49; 29%), inexperience of a clinical trial site is the primary trigger (12 of 14; 86%), followed by routine monitoring (10 of 14; 71%). Sufficient funds allocated to monitoring and expertise and training in onsite monitoring were reported as the most common enablers to onsite monitoring; the associated cost is the main barrier to onsite monitoring (Fig. 1, Supplementary Table 2).

Amongst all those who responded, 75% (71 of 95) had an onsite monitoring visit as part of an academic led clinical trial, with the top three reasons for the monitoring visit: source data verification (66 of 71; 93%), assessing compliance with the informed consent process (58 of 71; 82%) and assessing regulatory documents and investigator site files (56 of 71; 79%). Across respondents, the main perceived advantages of onsite monitoring are improved data quality and improved protocol adherence, while increased workload for both the coordinating site and study site are the main perceived disadvantages (Supplementary Table 3).

### Remote monitoring

**Fig. 2:**
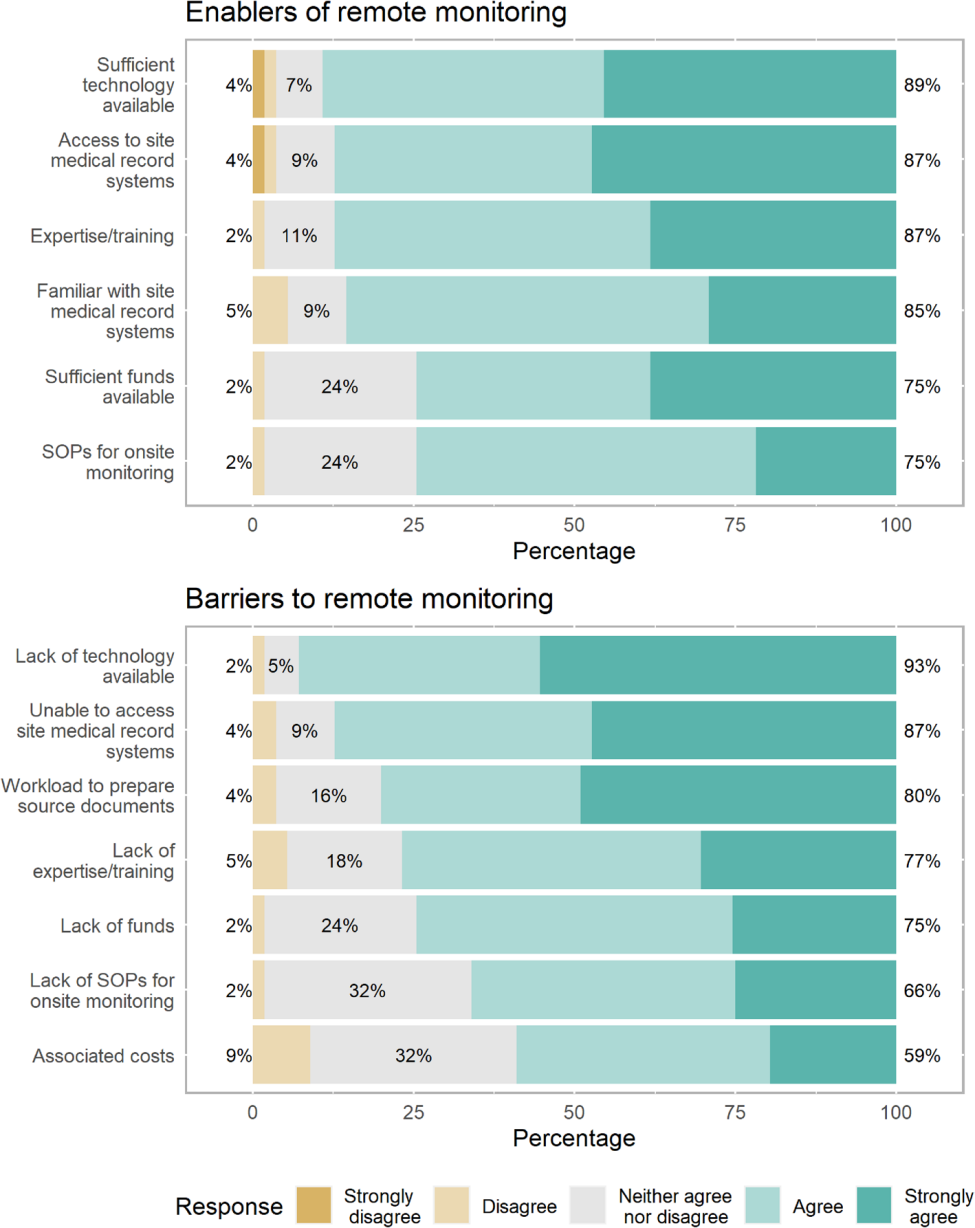
Perceived enablers and barriers to performing remote monitoring

For respondents conducting academic led clinical trials (*N* = 70), remote monitoring is performed by the respondent’s research unit 53% (30 of 57 responses) of the time, having been used by most units for more than one year (15 of 30; 50%). Where remote monitoring is conducted, the main study aspect assessed is source data verification (20 of 30; 67%). Remote monitoring is used to supplement onsite monitoring 87% (26 of 30) of the time, and replace onsite monitoring 70% (21 of 30) of the time. The main tools used to access study documents remotely are screening sharing software (e.g. Zoom, Microsoft Teams) (16 of 30; 53%), and document upload to online storage platforms (15 of 30; 50%). Ability to obtain remote access to medical records and technology to support remote monitoring are the main enablers to remote monitoring, while lack of technology to support remote monitoring is the main barrier (Fig. 2, Supplementary Table 4). Among all respondents, 46% were remotely monitored as part of an academic led clinical trial, having the primary purpose of source data verification. Across respondents, the main perceived advantages of remote monitoring are reduced monitoring costs and improved data quality, while increased technology requirements is the main disadvantage (Supplementary Table 5).

For those conducting source data verification as part of onsite or remote monitoring (46 of 70; 65%), 100% of source data is verified for consent (32 of 44; 73%), primary outcomes (30 of 44; 68%) and serious adverse events (32 of 44; 73%). The percentage of source data verification for selected case report form data is mainly predefined in the monitoring plan (26 of 46; 57%), and is conducted most commonly using a database-based tool (28 of 46; 61%).

### Centralised monitoring

**Fig. 3:**
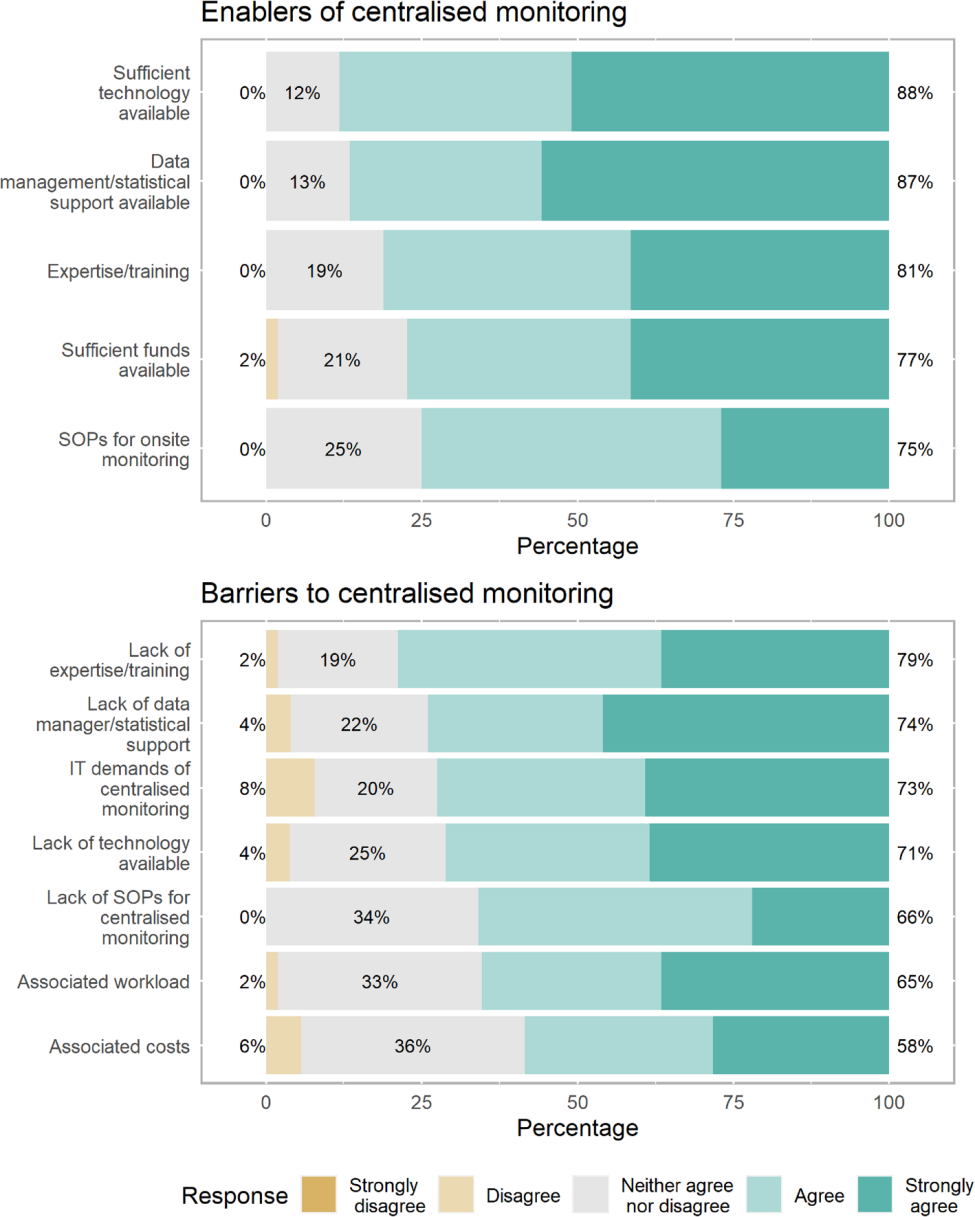
Perceived enablers and barriers to performing centralised monitoring

Centralised monitoring is conducted 33% (19 of 58) of the time. Where centralised monitoring is conducted, the main study aspects assessed are missing or invalid data (16 of 19; 84%) and rates of adverse events (14 of 19; 74%). It is used to guide, target or supplement onsite monitoring visits 79% (15 of 19) of the time, and sometimes used to replace onsite monitoring 32% (6 of 19) of the time. A computer program written for each trial is the main tool used for centralised monitoring (7 of 19; 37%), with tools primarily being developed by the research unit (11 of 19; 58%). Technology to support centralised monitoring along with support from data managers and statisticians are key enablers. Lack of education and training in centralised monitoring is the primary barrier (Fig. 3; Supplementary Table 6).

Among all respondents, the main perceived advantages of centralised monitoring are earlier identification of data quality issues and improved data quality, while the main perceived disadvantage is increased information technology demands (Supplementary Table 7).

### Impact of COVID-19 pandemic

The COVID-19 pandemic has an impact on the conduct of monitoring for 82% (45 of 55) of respondents from units that coordinate academic led clinical trials. Monitoring was impacted by increasing remote (25 of 45; 56%) and reducing onsite (29 of 45; 64%) monitoring.

### Association of respondent characteristics with key monitoring activities

Exploratory analysis revealed that New Zealand respondents, and those that had been working in clinical trials for ≤ 6 years, were less likely to develop a monitoring plan for a clinical trial (Supplementary Table 8). Respondents working primarily in adult research may be more likely to develop a monitoring plan, compared with their paediatric counterparts. Risk assessments tended to be undertaken more frequently by respondents who had attended face-to-face GCP training or monitoring-specific training.

## Discussion

Appropriate monitoring of accruing research data is a crucial aspect of high-quality clinical trials. Although a significant portion of the cost of a clinical trial is associated with data monitoring source documentation errors consistently rank as a deficiency in clinical trial inspections [28, 29]. Resources associated with clinical trials sponsored by industry permits extensive and time-consuming approaches to monitoring, however academic-led clinical trials do not generally share this luxury. Our research sought to understand the current state of monitoring practices in an emerging area of clinical trial activity that is generally not associated with industry-led trials.

Our survey indicated that the majority of research staff working in units undertaking clinical trials in critical care research in ANZ develop monitoring plans, however more than a third are not basing these plans on a risk assessment, as advocated by ICH-GCP, in contrast with findings from the UK where nearly all CTUs reported using risk assessments [20], but with similar findings to a European study [21] and in Ireland [22]. While there are recognised advantages that risk-based methodology improves data accuracy and patient safety, a lack of expertise in undertaking the risk assessment was a barrier for almost half of the respondents who don’t currently use this method. Despite a high level of uptake of ICH-GCP training, this aspect of the ICH-GCP guidelines is not well covered in training opportunities.

While onsite monitoring visits are still a common component of a monitoring plan, remote and centralised monitoring are gaining importance and regularity. As a result of the COVID-19 pandemic, onsite monitoring was significantly decreased, while the use of remote monitoring increased for more than 50% of respondents. However, there remains significant shared challenges relating to remote access to medical records to enable remote monitoring, despite a move towards electronic health records, which need to be addressed to enable remote monitoring to occur efficiently. Similarly, increased technological and statistical support is required to enhance remote and centralised monitoring. These are not challenges faced only in the critical care setting, with these findings applicable across a diverse range of research areas. Addressing these barriers will support the move towards a reduction in the total monitoring costs across clinical trials, while maintaining, or enhancing, the data quality.

This is the first study to report on the use of risk-based monitoring in Australia and New Zealand. While it is limited to the critical care setting, the findings are likely generalisable to other settings where clinical trials are predominantly academic-led, and not industry sponsored. The professional networks that distributed the survey were both clinical and research focussed, leading to a low number of responses compared with the number of email addresses it was distributed to. However, with email addresses in the targeted research groups totalling approximately 1200 (significant overlap in these groups would further significantly reduce the number of unique recipients), and the authors knowledge of this research community, 70 individual respondents is a reasonable representation of researchers closely involved in critical care research in the region. We chose to survey individuals, rather than research groups, so there may be overlap in responses from multiple respondents in one research unit. This approach was chosen as our experience indicates that within a research group the level of understanding and practice may differ between staff and projects. Our piloting of the survey was limited, however the reviewers were multidisciplinary, and components of the study had derived from studies previous published.

We support the call by Love et al. [24] to elevate the requirement for a clinical trial monitoring plan to the same status as a protocol and statistical analysis plan. Presently, there are limited resources and guidance documents to provide direction on the processes involved with developing a robust monitoring plan, including risk assessment and implementation of associated risk mitigation and monitoring strategies. With two-thirds of respondents expressing a desire for training specific to clinical trials monitoring, there are opportunities to collaborate across jurisdictions to develop training programs relating to the development of RBM plans. Coupled with enhanced technological and statistical solutions, the implementation of RBM in the ANZ critical care setting has the potential to be greatly enhanced.

## Supplementary Information

**Additional file 1:** **Table S1**. Characteristics of survey respondents. **Table S2**. Perceived enablers and barriers to performing onsite monitoring. **Table S3**. Reported advantages and disadvantages of onsite monitoring (*N* = 70). **Table S4**. Perceived enablers and barriers to performing remote monitoring. **Table S5**. Reported advantages and disadvantages of remote monitoring. **Table S6**. Perceived enablers and barriers to performing centralised monitoring. **Table S7**. Reported advantages and disadvantages of centralised monitoring. **Table S8**. Association of respondent characteristics with development of a monitoring plan and undertaking a risk assessment.

## Data Availability

The datasets generated and/or analysed during the current study are not publicly available as this was not pre-specified in our approved protocol, but are available from the corresponding author on reasonable request.
