# Supplementary material for: Research monitoring practices in critical care research: a survey of current state and attitudes
Source: BMC Med Res Methodol. 2022 Mar 21;22:74. doi: 10.1186/s12874-022-01551-7 (PMC8935263; doi:10.1186/s12874-022-01551-7)
Supplement: Supplementary file 1 — Additional file 1: Table S1. Characteristics of survey respondents. Table S2. Perceived enablers and barriers to performing onsite monitoring. Table S3. Reported advantages and disadvantages of onsite monitoring (N = 70). Table S4. Perceived enablers and barriers to performing remote monitoring. Table S5. Reported advantages and disadvantages of remote monitoring. Table S6. Perceived enablers and barriers to performing centralised monitoring. Table S7. Reported advantages and disadvantages of centralised monitoring. Table S8. Association of respondent characteristics with development of a monitoring plan and undertaking a risk assessment. [file 12874_2022_1551_MOESM1_ESM.docx]

**Title: Research monitoring practices in critical care research: a survey of current state and attitudes**

**Le Marsney et al**

Supplementary Table 1. Characteristics of survey respondents

| **Characteristic** | **N=118**  **n (%)** |
| --- | --- |
| ***Research Unit*** | |
| *Research Unit Affiliation** |  |
| Hospital | 103 (87.3) |
| University | 49 (41.5) |
| Other | 7 (5.9) |
| *Country* |  |
| Australia | 102 (86.4) |
| New Zealand | 16 (13.6) |
| *Patient Group* |  |
| Paediatric | 38 (32.2) |
| Adult | 80 (67.8) |
| *Additional Area/s of Research** |  |
| Emergency Medicine | 44 (37.3) |
| Intensive Care Medicine | 53 (44.9) |
| Anaesthetic Medicine | 33 (28.0) |
| Operating Room Medicine | 17 (14.4) |
| Other | 12 (10.2) |
| *Clinical Trial Types** |  |
| Academic-led | 105 (89.0) |
| Industry-led | 53 (44.9) |
| International | 101 (85.6) |
| Single-site | 73 (61.9) |
| Multi-site | 114 (96.6) |
| ***Respondent*** | |
| *Clinical Trial Types** |  |
| Academic-led | 103 (87.3) |
| Industry-led | 47 (39.8) |
| International | 96 (81.4) |
| Single-site | 71 (60.2) |
| Multi-site | 112 (94.9) |
| *Primary Role* |  |
| Principal Investigator | 23 (19.5) |
| Research Coordinator | 63 (53.4) |
| Research Nurse | 16 (13.6) |
| Study Monitor | 5 (4.2) |
| Data Manager | 2 (1.7) |
| Pharmacist | 1 (0.9) |
| Other | 8 (6.8) |
| *Years in Clinical Trials* |  |
| <1 year | 3 (2.5) |
| 1-3 years | 29 (24.6) |
| 4-6 years | 26 (22.0) |
| >6 years | 60 (50.9) |
| *Highest Level of Education* |  |
| Undergraduate Degree | 11 (9.4) |
| Postgraduate Degree | 89 (76.1) |
| Doctorate | 17 (14.5) |
| *Clinical Trials Training** |  |
| Good Clinical Practice – Face-to-face | 89 (75.4) |
| Good Clinical Practice – Online | 102 (86.4) |
| Monitoring specific training | 43 (36.4) |
| Other | 18 (15.3) |
| None of the above | 2 (1.7) |

* one or more responses could be selected

Supplementary Table 2. Perceived enablers and barriers to performing onsite monitoring

|  | **Strongly agree**  **n (%)** | **Agree**  **n (%)** | **Neither agree nor disagree**  **n (%)** | **Disagree**  **n (%)** | **Strongly disagree**  **n (%)** |
| --- | --- | --- | --- | --- | --- |
| **Enablers** | | | | | |
| Expertise and training in onsite monitoring | 27 (44) | 26 (43) | 7 (12) | 1 (2) | 0 |
| Familiarity with site-specific medical record systems | 19 (31) | 27 (44) | 11 (18) | 4 (7) | 0 |
| Sufficient funds allocated to monitoring in the study budget | 35 (57) | 21 (34) | 4 (7) | 1 (2) | 0 |
| Ability to organise appropriate level of access to site-specific medical record systems | 25 (40) | 26 (43) | 9 (15) | 1 (2) | 0 |
| Standard Operating Procedures for onsite monitoring | 15 (25) | 31 (50) | 15 (25) | 0 | 0 |
| Close proximity of study sites to coordinating site | 18 (13) | 21 (34) | 22 (36) | 10 (16) | 0 |
| **Barriers** | | | | | |
| Lack of expertise and training in onsite monitoring | 19 (31) | 28 (46) | 11 (18) | 2 (3) | 1 (2) |
| Cost associated with onsite monitoring | 22 (36) | 30 (49) | 7 (12) | 2 (3) | 0 |
| Workload associated with onsite monitoring | 23 (38) | 24 (39) | 7 (12) | 7 (12) | 0 |
| Unfamiliarity with site-specific medical record systems | 11 (18) | 22 (37) | 16 (27) | 11 (18) | 0 |
| Lack of funds allocated to monitoring in the study budget | 23 (38) | 28 (46) | 7 (12) | 3 (5) | 0 |
| Inability to organise appropriate level of access to site-specific medical record systems | 16 (26) | 27 (44) | 14 (23) | 3 (5) | 1 (2) |
| Lack of Standard Operating Procedures for onsite monitoring | 10 (17) | 28 (47) | 19 (32) | 3 (5) | 0 |
| Study sites not in close proximity to coordinating site | 10 (16) | 19 (31) | 19 (31) | 10 (16) | 3 (5) |

Supplementary Table 3. Reported advantages and disadvantages of onsite monitoring

| **Advantage** | **n (%)** |
| --- | --- |
| Improved data quality | 88 (75) |
| Improved protocol adherence | 80 (68) |
| Improved understanding of the study site environment | 76 (64) |
| Improved patient safety | 61 (52) |
| Improved recruitment rates | 32 (27) |
| Reduced monitoring costs | 6 (5) |
| Other | 2 (2) |
| None | 1 (1) |
| Not sure | 0 (0) |
| **Disadvantage** | **n (%)** |
| Increased workload for staff at study sites | 57 (48) |
| Increased workload for staff at the coordinating site | 56 (48) |
| Increased monitoring costs | 51 (43) |
| Increased IT demands | 28 (24) |
| Delayed identification of data quality issues | 22 (19) |
| Increased expertise and training required | 12 (10) |
| None | 9 (8) |
| Not sure | 3 (3) |
| Other | 0 (0) |

Supplementary Table 4. Perceived enablers and barriers to performing remote monitoring

|  | **Strongly agree**  **n (%)** | **Agree**  **n (%)** | **Neither agree nor disagree**  **n (%)** | **Disagree**  **n (%)** | **Strongly disagree**  **n (%)** |
| --- | --- | --- | --- | --- | --- |
| **Enablers** | | | | | |
| Expertise and training in remote monitoring | 21 (38) | 27 (49) | 6 (11) | 1 (2) | 0 |
| Familiarity with site-specific medical record systems | 16 (29) | 31 (56) | 5 (9) | 3 (6) | 0 |
| Sufficient funds allocated to remote monitoring in the study budget | 21 (38) | 20 (36) | 13 (24) | 1 (2) | 0 |
| Ability to organise appropriate level of remote access to site-specific medical record systems | 26 (47) | 22 (40) | 5 (9) | 1 (2) | 1 (2) |
| Standard Operating Procedures for remote monitoring | 12 (22) | 29 (53) | 13 (24) | 1 (2) | 0 |
| Sufficient technology to support remote monitoring | 25 (46) | 24 (44) | 4 (7) | 1 (2) | 1 (2) |
| **Barriers** | | | | | |
| Lack of expertise and training in remote monitoring | 17 (30) | 26 (46) | 10 (18) | 3 (5) | 0 |
| Cost associated with remote monitoring | 11 (20) | 22 (39) | 18 (32) | 5 (9) | 0 |
| Workload associated with preparation of source documents by study site for remote monitoring | 27 (49) | 17 (31) | 9 (16) | 2 (4) | 0 |
| Lack of funds allocated to remote monitoring in the study budget | 14 (26) | 27 (49) | 13 (24) | 1 (2) | 0 |
| Inability to organise appropriate level of remote access to site-specific medical record systems | 26 (47) | 22 (40) | 5 (9) | 2 (4) | 0 |
| Lack of Standard Operating Procedures for remote monitoring | 14 (25) | 23 (41) | 18 (32) | 1 (2) | 0 |
| Lack of technology to support remote monitoring | 31 (55) | 21 (38) | 3 (5) | 1 (2) | 0 |

Supplementary Table 5. Reported advantages and disadvantages of remote monitoring

| **Advantage** | **n (%)** |
| --- | --- |
| Reduced monitoring costs | 64 (54) |
| Improved data quality | 51 (43) |
| Improved protocol adherence | 35 (30) |
| Improved patient safety | 27 (23) |
| Improved recruitment rates | 12 (10) |
| Not sure | 9 (8) |
| None | 3 (3) |
| Other | 1 (1) |
| **Disadvantage** | **n (%)** |
| Increased IT demands | 44 (37) |
| Increased workload for staff at study sites | 40 (34) |
| Increased workload for staff at the coordinating site | 36 (31) |
| Delayed identification of data quality issues | 27 (23) |
| Increased expertise and training required | 19 (16) |
| Not sure | 10 (9) |
| None | 9 (8) |
| Increased monitoring costs | 8 (7) |
| Other | 3 (3) |

Supplementary Table 6. Perceived enablers and barriers to performing centralised monitoring

|  | **Strongly agree**  **n (%)** | **Agree**  **n (%)** | **Neither agree nor disagree**  **n (%)** | **Disagree**  **n (%)** | **Strongly disagree**  **n (%)** |
| --- | --- | --- | --- | --- | --- |
| **Enablers** | | | | | |
| Expertise and training in centralised monitoring | 22 (42) | 21 (40) | 10 (19) | 0 | 0 |
| Sufficient funds allocated to centralised monitoring in the study budget | 22 (42) | 19 (36) | 11 (21) | 1 (2) | 0 |
| Standard Operating Procedures for centralised monitoring | 14 (27) | 25 (48) | 13 (25) | 0 | 0 |
| Sufficient technology to support centralised monitoring | 26 (51) | 19 (37) | 6 (12) | 0 | 0 |
| Sufficient support from data managers and statisticians | 29 (56) | 16 (31) | 7 (14) | 0 | 0 |
| **Barriers** | | | | | |
| Lack of education and training in centralised monitoring | 19 (37) | 22 (42) | 10 (19) | 1 (2) | 0 |
| Cost associated with centralised monitoring | 15 (28) | 16 (30) | 19 (36) | 3 (6) | 0 |
| IT demands of centralised monitoring | 20 (39) | 17 (33) | 10 (20) | 4 (8) | 0 |
| Workload associated with centralised monitoring | 19 (37) | 15 (29) | 17 (33) | 1 (2) | 0 |
| Lack of technology to support centralised monitoring | 20 (39) | 17 (33) | 13 (25) | 2 (4) | 0 |
| Lack of support from data managers and statisticians | 23 (46) | 14 (28) | 11 (22) | 2 (4) | 0 |
| Lack of Standard Operating Procedures for centralised monitoring | 11 (22) | 22 (44) | 17 (34) | 0 (0) | 0 |

Supplementary Table 7. Reported advantages and disadvantages of centralised monitoring

| **Advantage** | **n (%)** |
| --- | --- |
| Earlier identification of data quality issues | 62 (53) |
| Improved data quality | 62 (53) |
| Improved efficiency and objectivity of onsite monitoring | 46 (39) |
| Reduced monitoring costs | 44 (37) |
| Improved protocol adherence | 40 (34) |
| Improved patient safety | 25 (21) |
| Improved recruitment rates | 9 (8) |
| Not sure | 7 (6) |
| Other | 0 (0) |
| None | 0 (0) |
| **Disadvantage** | **n (%)** |
| Increased IT demands | 37 (31) |
| Increased workload for staff at the coordinating site | 34 (29) |
| Increased workload for staff at study sites | 29 (25) |
| Increased expertise and training required | 28 (24) |
| Not sure | 19 (16) |
| None | 8 (7) |
| Other | 2 (2) |

Supplementary Table 8. Association of respondent characteristics with development of a monitoring plan and undertaking a risk assessment.

| **Characteristic** | | **Development of a Monitoring Plan** | | | **Undertaking a Risk Assessment** | | |
| --- | --- | --- | --- | --- | --- | --- | --- |
|  |  | **No**  **N=14** | **Yes**  **N=56** | **Odds Ratio (95% Confidence Interval)** | **No**  **N=23** | **Yes**  **N=33** | **Odds Ratio (95% Confidence Interval)** |
|  |  | **n (%)** | **n (%)** |  | **n (%)** | **n (%)** |  |
| Institution type | Hospital | 13 (93) | 45 (80) | 0.31 (0.034, 2.67) | 19 (83) | 26 (79) | 0.78 (0.20, 3.06) |
|  | University | 7 (50) | 29 (52) | 1.07 (0.33, 3.47) | 12 (52) | 17 (52) | 0.98 (0.34, 2.83) |
|  | Other | 0 (0) | 5 (9) | - | 1 (4) | 4 (12) | 3.03 (0.32, 29.09) |
| Country | Australia | 9 (64) | 53 (95) | *Reference* | 23 (100) | 30 (91) | - |
|  | New Zealand | 5 (36) | 3 (5) | 0.10 (0.021, 0.50) | 0 (0) | 3 (9) | - |
| Patient group | Paediatric | 9 (64) | 22 (39) | *Reference* | 8 (35) | 14 (42) | *Reference* |
|  | Adult | 5 (36) | 34 (61) | 2.78 (0.82, 9.40) | 15 (65) | 19 (58) | 0.72 (0.24, 2.18) |
| Types of research | Academic-led | 13 (93) | 52 (93) | 1 (0.10, 9.72) | 21 (91) | 31 (93) | 1.48 (0.19, 11.32) |
|  | Industry | 7 (50) | 26 (46) | 0.87 (0.27, 2.80) | 12 (52) | 14 (42) | 0.68 (0.23, 1.97) |
|  | International | 12 (86) | 50 (89) | 1.39 (0.25, 7.76) | 21 (91) | 29 (88) | 0.69 (0.12, 4.13) |
|  | Single-site | 10 (71) | 39 (70) | 0.92 (0.25, 3.34) | 15 (65) | 24 (73) | 1.42 (0.45, 4.50) |
|  | Multi-site | 14 (100) | 53 (95) | - | 22 (96) | 31 (94) | 0.70 (0.060, 8.26) |
| Trial role | Research Co-ordinator | 8 (57) | 21 (38) | *Reference* | 8 (35) | 13 (39) | *Reference* |
|  | Principal Investigator | 3 (21) | 16 (29) | 2.03 (0.46, 8.91) | 8 (35) | 8 (24) | 0.62 (0.16, 2.30) |
|  | Other | 3 (21) | 19 (34) | 2.41 (0.56, 10.44) | 7 (30) | 12 (36) | 1.05 (0.29, 3.80) |
| Trial experience | <6 years | 2 (14) | 35 (63) | 0.10 (0.020, 0.49) | 13 (57) | 22 (67) | 0.65 (0.22, 1.95) |
|  | >6 years | 12 (86) | 21 (38) | *Reference* | 10 (43) | 11 (33) | *Reference* |
| Training | GCP - Face-to-face | 9 (65) | 41 (73) | 1.52 (0.44, 5.26) | 14 (61) | 27 (82) | 2.89 (0.86, 9.78) |
|  | GCP - Online | 13 (93) | 50 (89) | 0.64 (0.071, 5.80) | 21 (91) | 29 (88) | 0.69 (0.12, 4.13) |
|  | Monitoring specific | 4 (29) | 22 (39) | 1.62 (0.45, 5.80) | 7 (30) | 15 (45) | 1.90 (0.62, 5.85) |

* GCP Good Clinical Practice
